# Human germline editing in the era of CRISPR-Cas: risk and uncertainty, inter-generational responsibility, therapeutic legitimacy

**DOI:** 10.1186/s12910-020-00487-1

**Published:** 2020-09-11

**Authors:** Sebastian Schleidgen, Hans-Georg Dederer, Susan Sgodda, Stefan Cravcisin, Luca Lüneburg, Tobias Cantz, Thomas Heinemann

**Affiliations:** 1 Faculty of Humanities and Social Sciences, Institute of Philosophy, FernUniversität in Hagen, Universitätsstraße 33, 58097 Hagen, Germany; 2 Faculty of Law, University of Passau, Innstraße 39, 94032 Passau, Germany; 3 Translational Hepatology and Stem Cell Biology, REBIRTH Center for Translational Regenerative Medicine, Department of Gastroenterology, Hepatology, and Endocrinology, Hannover Medical School, Carl-Neuberg-Str. 1, 30625 Hanover, Germany; 4 Faculty of Nursing Science, University of Philosophy and Theology Vallendar, Pallottistraße 3, 56179 Vallendar, Germany

**Keywords:** Germline therapy, Human embryos, Therapeutic legitimization, Responsibility for future generations, Risks

## Abstract

**Background:**

Clustered Regularly Interspaced Short Palindromic Repeats-associated (CRISPR-Cas) technology may allow for efficient and highly targeted gene editing in single-cell embryos. This possibility brings human germline editing into the focus of ethical and legal debates again.

**Main body:**

Against this background, we explore essential ethical and legal questions of interventions into the human germline by means of CRISPR-Cas: How should issues of risk and uncertainty be handled? What responsibilities arise regarding future generations? Under which conditions can germline editing measures be therapeutically legitimized? For this purpose, we refer to a scenario anticipating potential further development in CRISPR-Cas technology implying improved accuracy and exclusion of germline transmission to future generations. We show that, if certain concepts regarding germline editing are clarified, under such conditions a categorical prohibition of one-generation germline editing of single-cell embryos appears not to be ethically or legally justifiable.

**Conclusion:**

These findings are important prerequisites for the international debate on the ethical and legal justification of germline interventions in the human embryo as well as for the harmonization of international legal standards.

## Background

Ever since the publication of Friedmann and Roblin’s article “Gene Therapy for Human Genetic Disease?” in 1972 [1], the possibility as well as permissibility of modifying human deoxyribonucleic acid (DNA) is subject to intense debates in ethics and law. Three problems regarding germline therapies have been consistently discussed in ethics and law: (i) questions of risk and uncertainty related to the technology and its application, (ii) interference with the human germline and responsibility towards future generations, and (iii) the legitimization of genome editing measures with regard to the concepts of therapy and enhancement. Since these questions point toward conceptual issues yet to be clarified, there is wide consensus that germline editing in human beings at present cannot be justified.

The introduction of CRISPR-Cas has stirred up again normative debates on human germline editing. This technology spread rapidly in biomedical research as it allows for a comparatively easy, efficient and precise targeted editing of the human genome [2, 3]. For this purpose, a CRISPR-associated protein 9 (Cas9) is guided by custom-made short ribonucleic acid (RNA)-sequences (guideRNAs) to specific genomic loci where it acts as molecular scissors inducing DNA breaks. The resulting DNA cleavage activates a cellular repair mechanism (non-homologous end-joining) that seeks to reassemble the clipped DNA ends. This results in rejoining the DNA ends but may also lead to, e.g., DNA insertions or deletions. However, when adding a defined DNA-repair template, an insertion of this sequence results in precise genetic edits at the given DNA-site (homology driven repair) [4, 5]. Exploiting these mechanisms, gene editing via CRISPR-Cas has become rapidly available for numerous approaches ranging from cell culture and in vivo applications to the manipulation of early human embryos. The first report of CRIPSR-Cas mediated editing of human embryos was published in 2016 [6, 7]. In November 2018, the birth of twin girls allegedly carrying an intentionally modified gene of the chemokine receptor type 5 (CCR5) was announced [8].

Besides modifying a few nucleotides of a given DNA sequence, CRISPR-Cas can also be used for introducing larger elements, i.e. transgene cassettes, to specific DNA loci. Such cassettes may consist of one or more genes controlled by independent promotor sequences, and could be used to co-introduce a DNA recombinase system that physically removes the gene(s) located in the cassette if this very promotor is activated [9, 10]. This may allow for the removal of a transgene cassette from, e.g., developing germ cells if it contains a recombinase system controlled by a germ cell-specific promotor. Such a design would confine the edited genome to the treated individual, leaving, however, future generations unaffected (so-called “one-generation germline therapy”).

In the following, we examine potential implications of the CRISPR-Cas technology for an evaluation of the three major ethical and legal problem complexes regarding human germline editing, i.e. questions of risk and uncertainty, inter-generational responsibility, and therapeutic legitimacy. For this purpose, we use cystic fibrosis (CF) as a clinical example for a frequent autosomal recessive genetically transmitted disease and re-analyze the ethical and legal arguments regarding genome editing in single-cell human embryos with CRISPR-Cas mediated treatment. This hypothetical situation can count as realistic insofar as it includes advances in the development of CRISPR-Cas that have so far not been accomplished but are currently intensively studied and may be available in the future [11, 12]. Using this approach, we show that if the accuracy of CRISPR-Cas mediated genome editing can be improved and germline transmission to future generations be excluded, the editing of human single-cell embryos appears to be no matter of categorical arguments, but rather one of safety aspects.

## Main text

### Risk and uncertainty

#### Ethical and legal arguments so far

CRISPR-Cas, although raising hopes and expectations regarding the safe and effective treatment of severe, hitherto incurable hereditary human diseases, has provoked intense ethical and legal debates with a view to possible risks associated with the technology. At present, CRISPR-Cas does not work sufficiently precise, leading to so-called off-target effects, i.e. unintended changes in non-target locations of the genome with unknown effects on treated cells [6, 13–15]. If applied in human single-cell embryos, it is stated from an ethical point of view, such edited embryos would bear unacceptable risks because of such off-target effects. On the other hand, it is argued that these risks would not speak against germline editing, but rather in favor of further research with the aim of risk minimization [16–18].

These arguments have been put forward in the context of germline interventions long before the advent of CRISPR-Cas. Risk assumptions prompted many national regulators to ban or restrict human germline modification. For example, the German legislature prohibited any artificial modification of germline cells [19] because, from the legislator’s perspective, any such treatment would, initially, require experiments on human beings [20]. Such experiments, however, would have to be considered irresponsible in view of potentially irreversible consequences for the involved individuals. Similarly, in the United States, the National Institutes of Health (NIH) based their decision not to fund “any use of gene-editing technologies in human embryos” [21] on the consideration that “[t]he concept of altering the human germline in embryos for clinical purposes” raises “serious and unquantifiable safety issues” [21].

Such decisions by national legislators or regulators^1^ may have been guided by requirements arising from constitutional law or international human rights law. In Germany, e.g., fundamental rights such as the right to life and physical integrity [23] are not only negative rights, but rather impose positive obligations on the government to protect human life and physical integrity against, e.g., risks arising from new technologies [24]. The European Court of Human Rights (ECtHR), for instance, has firmly established the doctrine of positive obligations arising from human rights [25]. On the other hand, the concept of positive obligations arising from fundamental rights has, for example, not gained acceptance in US constitutional law doctrine or jurisprudence [26].

With the recent advancements of the CRISPR-Cas technology, however, it seems within reach that risks for the life and health of human embryos as well as uncertainties regarding long-term effects for edited embryos and their descendants arising from germline interventions can be minimized to an acceptable level.^2^ Ever since its discovery, great effort has been put into further improving the accuracy of CRISPR-Cas [28]. In the following scenario 1, we assume that due to substantial improvements in CRISPR-Cas technology the risks of accidentally altering the germline could be considered negligible.

##### Scenario 1: gene editing at the endogenous CF-related gene locus

**Fig. 1:**
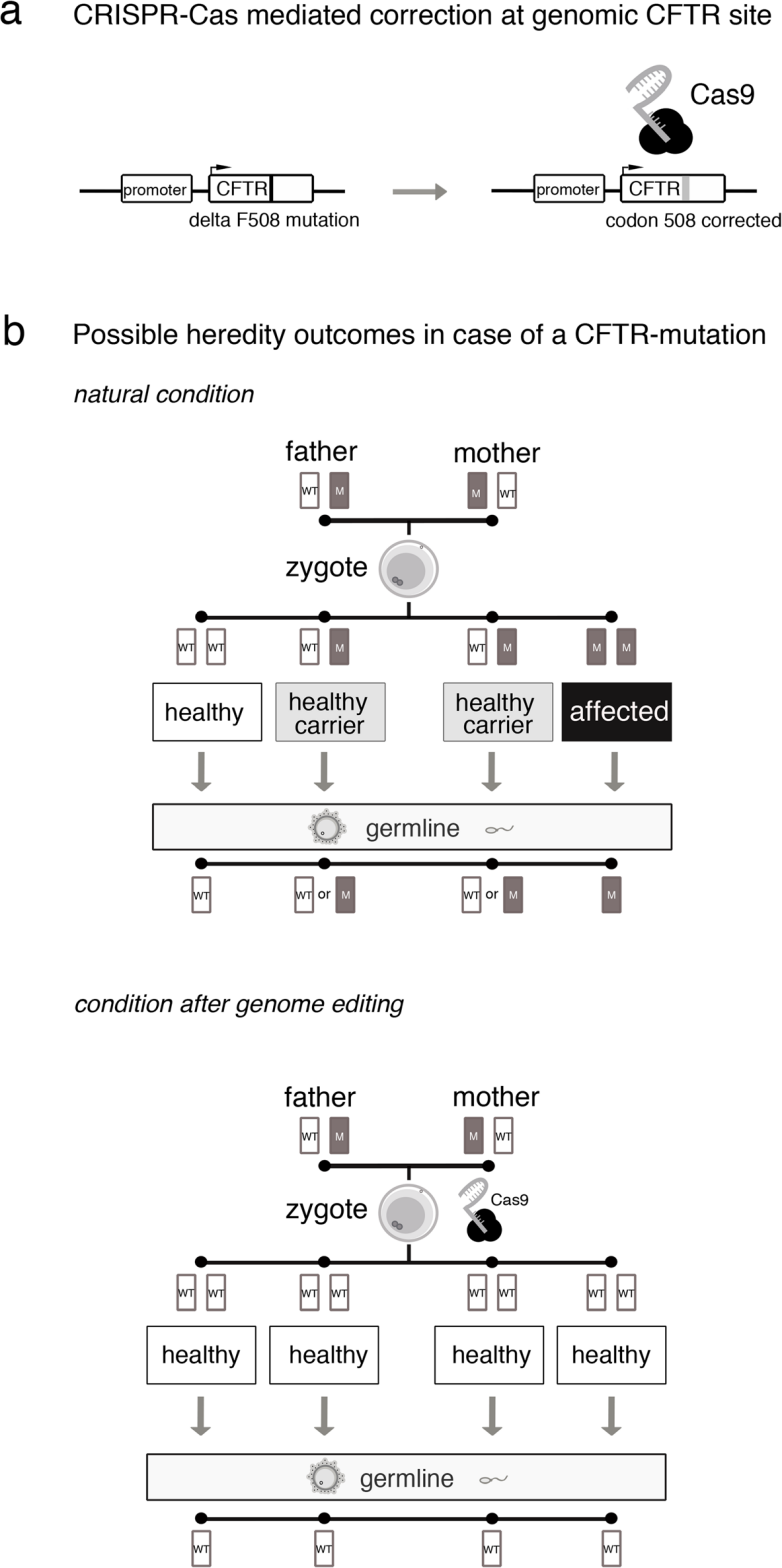
Scenario 1. Hereditary outcomes of CRISPR-Cas mediated correction at the CFTR site (WT: wild-type allele; M: mutant allele)

In scenario 1, CRISPR-Cas is used to edit the CF-underlying defect at the endogenous gene locus (cystic fibrosis transmembrane conductance regulator, CFTR) in all in vitro generated human embryos descending from a CF-carrier couple (see Fig. 1a). As such single-cell embryos cannot be diagnosed without being destroyed, all alleles, whether mutation carrying or not, are remodeled to the wildtype sequence of the functionally normal gene. Editing the endogenous gene locus entails that all correlated cells of the developing embryo produce the wildtype form of CFTR and, thus, that the embryo and all future offspring develop healthy (see Fig. 1b).

When in vitro fertilization procedures including intracytoplasmic sperm injection (ICSI) or other sophisticated techniques are applied in couples suffering from infertility, a given amount of genetic damage of the isolated gametes as well as of the developing embryo during in vitro propagation needs to be considered, even if no genomic interventions are pursued. Scenario 1 rests on the core assumption that due to improvements of the CRISPR-Cas technology risks arising from off-target effects of the genomic intervention would have only a minor impact. This means that the overall risk of genome editing interventions would not significantly deviate from the risks arising from the mutation rate of routinely used complex procedures in assisted reproduction medicine, defined as the rate of genetic sequence variations during fertilization and the first steps of embryonic development. If this risk level was considered sufficiently small, our assumption undermines, first, arguments concerning risks for off-target effects and, second, arguments in favor of preferring preimplantation genetic diagnosis (PGD) and subsequent selection of embryos as already available safe alternative over genome editing in human embryos.

#### Remaining concerns

Assuming the overall risk of the intervention in scenario 1 is scoring within the risks arising from the mutation rate of normal reproduction or already established assisted reproduction procedures, the question arises of whether existing regulations referring to unacceptable risks of germline interventions would have to be adjusted. For instance, both the German Federal Constitutional Court [29] and the ECtHR [30] have held that governments are obliged to observe future development in the sciences. Accordingly, the German legislator as well as the contracting parties to the European Convention of Human Rights may be under the obligation to revisit their national prohibitions of human germline modifications and, ultimately, to revoke the ban or allow for exceptions if prior assumptions of risk are refuted by new scientific insights or technological progress. In particular, with regard to scenario 1, it could be argued that the positive obligation to take life and health protecting measures is not triggered any longer by gene editing, if the rate of unintended effects of genome editing by use of CRISPR-Cas is within the mutation rate of assisted reproduction during complex in vitro fertilization procedures. Consequently, as far as the prohibition of human germline editing is based on the argument of unacceptable risks to human life and health of the embryo and its offspring, national legislators might be legally obliged to revoke absolute bans on human germline interventions. In addition, one may hold that this is supported by international human rights law. According to Article 15(1)(b) of the International Covenant on Economic, Social and Cultural Rights (ICESCR) [31], states must recognize everyone’s right “to enjoy the benefits of scientific progress and its applications”. Hence, if our core assumptions proved true, i.e. the overall risk of genome editing interventions did not significantly add to the risks arising from the mutation rate of assisted reproduction during in vitro fertilization procedures, and, hence, this form of therapy was scientifically feasible, states could be considered to be under the positive obligation to make such therapies available to patients. Retaining a prohibition of germline therapies might, furthermore, also be regarded as a violation of everyone’s right “to the enjoyment of the highest attainable standard of physical and mental health” (Article 12(1) ICESCR).

From an ethical point of view, the question arises why, if at all, any of the risks related to the technical intervention should be considered, provided that they score within the risks occurring in assisted reproduction during in vitro fertilization procedures. On this view, the assumption reflects the frequently presented argument that reducing known risks to a certain degree would make the application of genome editing measures unproblematic [32]. However, what this calls for is a clarification of the underlying epistemic as well as normative values. What is required, both in ethics and law, are adequate points of reference for regarding the overall risk, e.g. the rate of unintended off-target effects, as sufficiently low. One possibility, as implied in scenario 1, consists in referring to the mutation rate occurring in assisted reproduction procedures already established as a morally and epistemically appropriate point of reference for determining risks of genome editing measures as sufficiently small. Another possibility would be to establish alternative thresholds for morally or legally irrelevant risks of harm, for instance the threshold of non-detectability of risk effects. Both approaches come at the cost of difficulties to be solved, e.g. of falling victim to the fallacy of regarding non-detectable effects as irrelevant [33].

Even if the overall risk of a CRISPR-Cas mediated germline intervention lies within the risks of already established assisted reproduction procedures, and is, therefore, considered acceptable, negative long-term consequences, in particular for the edited embryo as well as its offspring, cannot be ruled out entirely. Hence, scenario 1 does not release from developing an ethically as well as legally acceptable strategy for coping with uncertainty.

In situations of uncertainty, an application of the so-called precautionary principle often is proposed. From a legal perspective, it may be safe to say that the principle entitles the legislator in a situation of scientific uncertainty to assume that harm is possible and to enact respective laws aiming at protection [34]. In philosophical terms, however, it is still largely unclear what the precautionary principle implies. Although manifold versions of the principle exist, Sandin has demonstrated that almost any version can be summed up under the abstract formula: “If there (1) is a threat, which is (2) uncertain, then (3) some kind of action (4) is mandatory” [35].

Thus, the principle comprises four dimensions. The threat (1) and uncertainty (2) dimensions establish the conditions of its application. The former specifies the potentially negative consequences which call for its application. The latter specifies the nature and extent of (scientific) uncertainty regarding the occurrence of these consequences in the sense of necessary conditions for its application. Threat (1) and uncertainty (2) dimensions are also reflected in legal doctrine on the precautionary principle. According to the European Commission, for instance, it applies only if scientists are able to identify, at least, the possibility of negative effects [34]. This is in line with the CJEU’s case-law [36] which explicitly held that “where, following an assessment of available information, the possibility of harmful effects on health is identified but scientific uncertainty persists, provisional risk management measures […] may be adopted” [37].^3^

The action (3) and command dimensions (4) are concerned with establishing precautionary measures. Whereas the former determines the required decision strategy, the latter defines the degree to which pursuing the proposed action is prescribed (e.g. as obligatory, permissible, etc.). In legal terms, e.g., the precautionary principle “justifies the adoption of restrictive measures, provided they are non-discriminatory and objective […and] proportionate and no more restrictive […] than is required to achieve the […] level of […] protection chosen” [37].

Ultimately, with a view to our scenario 1 and the assumption of the interventions’ overall risk lying within the mutation rate of established assisted reproduction procedures, all four dimensions of the precautionary principle would have to be specified to make the principle applicable.

### Responsibility towards future generations

#### Ethical and legal arguments so far

Even if risks and uncertainties associated with genome editing in single-cell embryos could be minimized to an acceptable level, the question remains of whether it is ethically and legally justified to transfer these genetic alterations to future generations. Several objections have been raised, e.g., that it would be morally unacceptable to artificially manipulate the germline as the “heritage of humanity”,^4^ or that human germline editing would, if not for therapeutic purposes, undermine future individuals’ autonomy [39].

None of such categorical arguments, whether they are considered valid or not, will be solved or explained away by making use of CRISPR-Cas as presented in scenario 1. However, CRISPR-Cas may also allow for “one-generation germline editing” leaving future generations unaffected.

##### Scenario 2: “one-generation germline therapy”

**Fig. 2:**
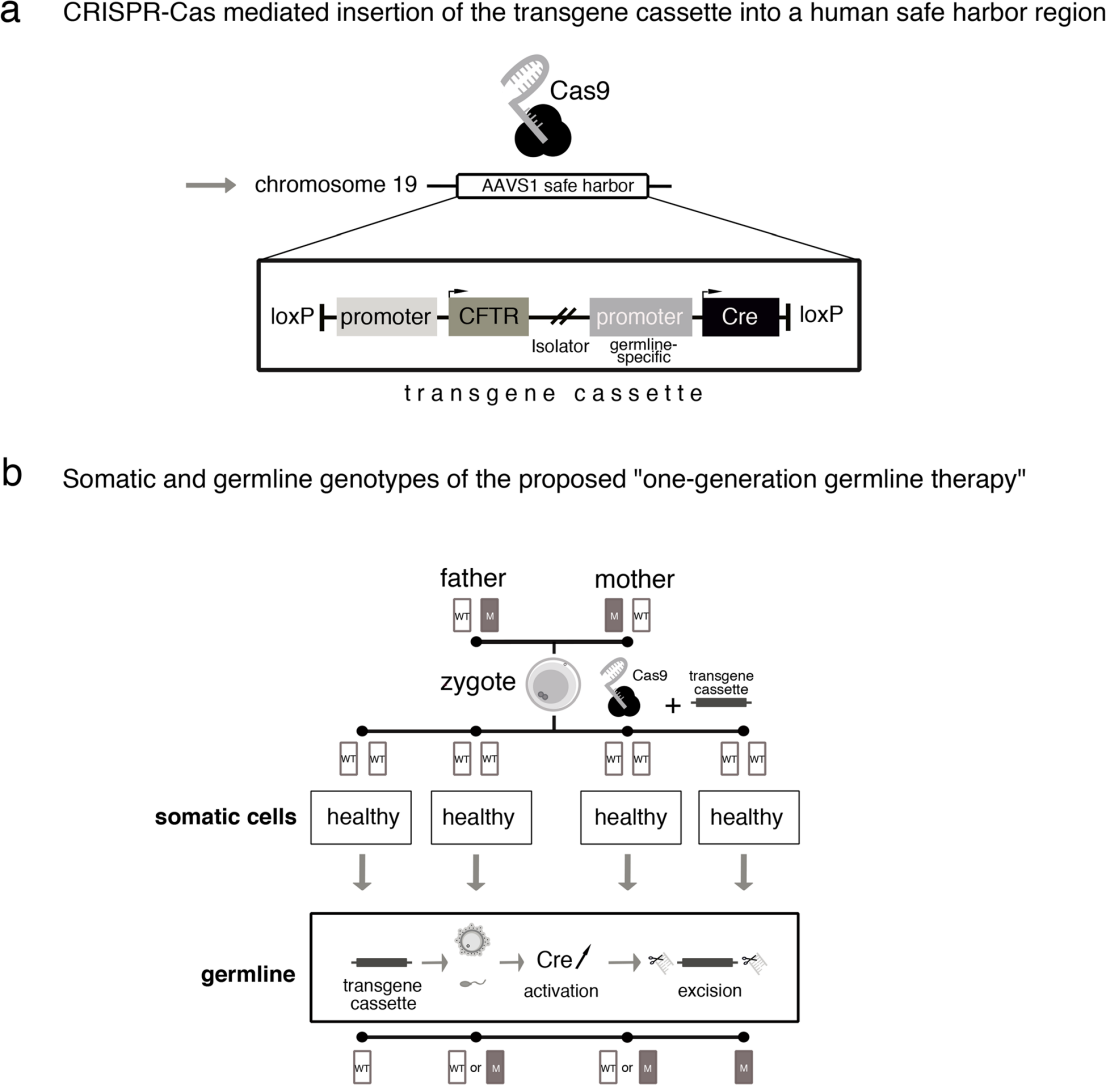
Scenario 2. “One-generation genome editing” (AAVS1: Adeno-Associated Virus Integration Site 1; WT: wild-type allele; M: mutant allele)

In scenario 2, embryos descending from a CF-carrier couple are not genetically modified at the endogenous CFTR gene. Rather, a more complex transgene cassette is introduced into a particular genomic locus. Such “safe harbor sites” allow for a stable integration of transgenes, while an interference with regulatory DNA-sequences or a transactivation of neighboring genes is avoided. The inserted transgene cassette can comprise more than one module, allowing for the expression of the CFTR wild-type sequence under the transcriptional control of its physiological promoter, and the expression of a DNA-recombinase under the transcriptional control of a germline-specific promoter (see Fig. 2a). Furthermore, the entire transgene cassette is flanked by specific recognition sites that allow for a removal of the cassette in all germ-line cells upon transcriptional activation of the DNA-recombinase. As a result, the endogenous CFTR gene locus is unaltered, while at the same time the additional wildtype-CFTR transgene is available in all somatic cells and the developing embryo is phenotypically cured of CF (see Fig. 2b). Its offspring, however, would not carry the transgene cassette as it is physically removed in all germ cells and, with the exception of a slight footprint in form of a few additional functionally inactive DNA nucleotides after recombination, remains unaffected from genome editing. As regards overall risk assessment, we assume for this scenario: first, the footprint in the genetic safe harbor site would not have functional consequences and, therefore, would not result in non-negligible risks. Second, future developments of gene editing tools will allow for highly efficient and precise insertion of transgenes and the overall risk of such genome editing interventions would not significantly deviate from the risks arising from the mutation rate of other complex procedures in assisted reproduction medicine.

#### Remaining concerns

Scenario 2 overcomes the normative problem of passing on genetic modifications in the germline of individual human beings to future generations and exposing future human beings, i.e. descendants of edited embryos, to unknown, possibly negative long-term effects without their consent, as well as affecting the human gene pool and, thus, humanity as a whole.

On the other hand, in scenario 2, the specific ethical issue is whether *not* passing on an edited genome to individuals of future generations needs justification. As the phenotype-correcting gene sequence is self-removing in germ cells, potential benefits of the intervention are limited to the edited embryo. Its descendants, however, are exposed to the risk of developing CF. To answer the question of whether this needs justification, first, the meaning of “exposing” descendants has to be clarified: is “exposing” to be understood intentionally and, hence, as an action calling for ethical evaluation? Only if this is answered in the affirmative it can be asked whether exposing future individuals to the risk of developing diseases like CF can be justified and, hence, be understood as a responsible action. Again, answering this question is possible, for instance, in view of the (potential) well-being of future individuals or the degree of naturalness of edited embryos.

From a legal perspective, at first sight, a “one-generation germline therapy” could be considered to be in conformity with the right to life and health of future human beings as long as adverse long-term effects of germline therapies cannot be ruled out. However, any legal restriction of germline therapies to “one-generation therapies” could conflict with the positive obligation of the State to protect human life and health [23, 24]. If germline therapy of, e.g., CF had to be considered safe for future generations according to science and technology, the legislator’s positive obligation to protect human life and health might transform into a legislative duty to permit such therapeutic germline modifications, e.g. for the treatment of CF patients, even beyond “one-generation” therapies [40]. In other words, with a view to its positive obligation to protect human life and health, the legislator might be precluded from permitting human germline editing under the restrictive technical conditions of scenario 2 only since, in that scenario, offspring of the edited embryo might still suffer from CF.

However, the conclusiveness of this inference depends on whether the State’s positive obligation to protect human life and health extends to future human beings, e.g. to future CF patients, at all. In addition, the problem of consent of future generations as well as of effects on the human gene pool and, hence, on humanity as such, would remain unsolved. In light of these remaining concerns, it might be considered not be contrary to the State’s positive obligation to protect human life and health, but within the State’s possibilities of decision-making, if the legislator confined the permissibility of germline therapies to “one-generation therapies” for the time being.

### Therapeutic legitimacy

Our prior considerations and scenarios suggest that categorical objections to human germline interventions may be overcome both scientific-technically and ethical-legally. If categorical prohibitions (to ban human germline editing completely) or dictates (to permit human germline editing in any case) cannot be convincingly established, the question arises under which conditions human germline editing might be considered legitimate in *individual* cases.

#### Ethical perspective: arguments and criteria for legitimizing germline editing interventions

The legitimacy of medical interventions into the physical integrity of human subjects usually relies on the informed and self-determined consent of the subjects concerned. Since consent cannot be obtained from single-cell embryos, the justification of genetic interventions is often discussed by reference to their (potential for) sufficient *therapeutic* benefit. Yet, looking at the germline editing procedures of scenarios 1 and 2 *as a whole,* it could be argued that they qualify as *preventive* rather than *therapeutic* measures. Ultimately, only in 25% of the embryos a genetic mutation would be corrected, 50% would lose carrier status while being phenotypically unchanged, and 25% would be left with the very same genetic sequence. If, however, *individual* embryos are considered legitimizing germline editing measures refers to the assumption that these embryos may benefit from the very genetic intervention. Hence, the (potential) therapeutic benefit is considered as key argument. Therefore, we discuss some of the presuppositions of focusing on therapeutic benefit in the context of germline editing measures as presented in our scenarios. We will conclude that, under certain conditions and with respect to individual embryos, it is possible to speak of such measures as *preemptive therapies*, i.e. as anticipative therapy without (knowledge of) existing pathologies (as known, e.g., from contexts like prophylactic mastectomy in cases of breast cancer gene [BRCA] mutations).

When referring to therapeutic benefit, it could be stated that legitimacy of human germline editing measures depends on a secured genetic diagnosis substantiating an individuals’ medical need (i.e. the expectation of her manifesting a relevant genetic disease), as well as the availability of an established gene therapy. On these grounds, referring to the publicly available information, the recently announced modification of CCR5 in human embryos with the aim of preventing the developing individuals from infection with human immunodeficiency virus (HIV) [8] could hardly be justified as therapeutic intervention. In fact, the developing embryos would not have been carrying a considerable risk for an HIV infection, if (washed) sperm was used from an HIV-positive father in an assisted reproduction setting. Hence, beside the fact that in this case gene editing was apparently performed without appropriate prior risk and safety assessments conducted by independent regulatory bodies, seemingly no medical need for the intervention was given [8].

However, germline editing does not seem to be justified by sole reference to (potential) therapeutic benefit in our scenarios either since no genetic diagnosis of CF can be performed in the individual single-cell embryos without destroying the respective zygotes. Thus, it is not knowable whether a certain embryo carries a diseased CF gene and, hence, would benefit from germline therapy. Rather, as shown in Fig. 1b, only probabilities can be given for the developing embryos either being healthy, healthy carriers of CF, or actually diseased. Consequently, it is questionable whether such genome editing interventions, without knowing the CF genotype in the individual single-cell embryos, can be considered *therapeutic* measures at all, or rather represent actions beyond therapeutic intention, and if so, how such actions may be legitimized. One way of answering this question would be to analyze the concepts of disease and diagnosis in terms of whether they may be adapted to situations like our scenarios and, hence, would allow for justifying genetic interventions in zygotes affected by *certain* diseases with a certain probability. A second type of argument could be based on re-analyzing regarding to whom genome editing interventions have to be justified or, in other words, who is to be regarded as patient: either the focus of justification is primarily on future parents (1), or it is primarily on the embryos or future children (2) [41]. If (1) is assumed, our scenarios seem to be similar to cases of selective reproduction [42] leading to considerations of reproductive autonomy, the value and meaning of genetic parenthood as well as possible alternatives to germline editing. If, however, (2) is held, according to an *alternative concept of therapy*, it is, certain conditions satisfied, plausible to label germline editing as (preemptive) therapy [41, 43].

Understanding germline editing in single-cell embryos as preemptive therapy in individuals requires two conditions being satisfied: First, the respective unedited zygote and the embryo resulting from the intervention must be regarded as ontologically identical (*identity condition*). Second, the intervention under consideration must promise (the potential for) sufficient overall benefit for an individual developing from an edited embryo (*benefit condition*).

The identity condition may seem trivial at first sight. It is, however, of particular importance in the context of germline editing in *single-cell* embryos, since CRISPR-Cas mediated changes in the genome of a zygote apply to all future cells in the developing embryo. Hence, the genetic make-up of the edited embryo differs in all subsequently developing cells from that of the unedited zygote as do the “normalized” physiological functions resulting from the genetic intervention. Therefore, germline edited individuals would have life conditions quite different from individuals developing from nonedited embryos. Against this background, it could be questioned (in contrast to most common medical contexts) whether the two entities under consideration are in fact identical. If they were not, however, the corresponding germline intervention could be neither regarded nor justified as a (preemptive) therapy. For any plausible concept of individual therapy necessarily relies on the assumption that individuals before and after an intervention are ontologically identical. Otherwise, it would be, e.g., logically impossible to justify an intervention in a certain individual by reference to its (potential) therapeutic benefit for this very individual.

The benefit condition, in turn, gains importance from the fact that (potential) benefits for individuals developing from edited embryos seem to be the only relevant *normative* aspect in the context of appropriate, i.e. justified medical decision-making regarding germline interventions in individual human embryos. For other normative claims usually relevant for medical decision-making, e.g. the consideration of patient autonomy through informed consent, are impossible to meet.

Following these considerations for an alternative concept of therapy, we may consider germline editing interventions as preemptive therapies with respect to their (potential) benefits for individuals developing from edited embryos (benefit condition), if the respective unedited zygotes and edited embryos are identical (identity condition). To support this claim, it has been suggested to specify the identity condition by reference to Parfit’s Origin View [43], according to which “[…] each person has this distinctive necessary property: that of having grown from the particular pair of cells from which this person in fact grew” [44]. Furthermore, it has been proposed to refine the benefit condition in view of a relative account of harm, according to which an individual is being harmed, if it is (possibly) worse off than it would have been in case of a certain action being taken [43]. Consequently, (sufficient) therapeutic benefit consists in avoiding such harm at the very least.

Parfit’s Origin View indeed suggests identity of the unedited zygotes and the edited embryos in our scenarios. Moreover, the individuals developing from the edited embryos can be held (possibly) worse off if the editing intervention had not been applied, and at least not harmed if the editing was applied (regardless of whether the edited zygote actually was healthy, a healthy carrier, or suffering from CF). Accordingly, it seems to be legitimate holding our scenarios as examples of (preemptive) therapy and, hence, to justify germline editing of a CFTR defect with regard to its (potential) therapeutic benefits.

However, both Parfit’s Origin View as well as relative accounts of harm have been contested [45–48]. As regards the former, it could be argued, e.g., that Parfit’s approach is ignoring decisive aspects of identity. In particular, interventions in the genome of embryos and their impact on the lives of edited individuals would make it difficult to understand unedited zygotes and edited embryos as qualitatively identical. As regards the latter, in view of our scenarios, the question arises, for instance, whether highly invasive genetic interventions in a human embryo can be adequately justified by reference to (sufficient) therapeutic benefit regarded as, at the very least, avoidance of harm. In contrast to many other medical interventions, e.g. oncological treatments, this is an issue precisely because a distinct diagnosis is lacking. The (necessary) renunciation of any reference to diagnosis in combination with the revised concept of therapeutic benefit in the alternative concept of therapy comes at the cost of therapeutically justifying germline editing in human single-cell embryos even though, like in our scenarios, 50% of the treated embryos would be phenotypically healthy without such intervention (and 25% even genotypically) (see Fig. 1b). Thus, the alternative concept of therapy, opponents could state, does not solve the problem of lacking diagnostic possibilities when deciding about germline interventions in single-cell embryos, but rather points toward the importance of diagnosis for justifying such measures.

In addition, it could be asked more generally whether the mere therapeutic intention to germline edit embryos *possibly* suffering from CF may be sufficient to adequately justify such interventions. These issue calls for further analysis of the alternative concept of therapy as well as the normative function of therapeutic intentions for an adequate justification of germline interventions like in our scenarios. Nevertheless, at least it seems possible that under certain theoretical assumptions both scenarios can be legitimized with regard to (sufficient) therapeutic benefit.

As regards scenario 1, however, the question arises whether passing on genomes to the offspring of edited embryos may also be justified as individual preemptive therapy. It is a trivial fact that the offspring of edited embryos cannot be identical with the unedited zygotes from which their parents developed. Hence, the identity condition is not satisfied for the offspring of edited embryos; passing on genomes to future individuals may not be justified by reference to a concept of preemptive therapy. Thus, insofar germline interventions can be justified as therapies at all, interventions as in scenario 2 seem preferable over interventions as in scenario 1.

In cases where germline editing measures are legitimate in view of their (potential) therapeutic benefit, the question arises of how to detect and deal with actually occurring side-effects in ethically acceptable ways. Postnatal monitoring of edited persons has been proposed, raising, however, important questions of whether, e.g., the individuals concerned are restrained regarding their autonomy, as well as organizational questions, for instance, of who (edited persons, their descendants) should be monitored in what time frames (5 years, 10 years, lifetime), and what monitoring measures would need to be applied by whom (state authorities, private institutions, parents) [49]. Here, too, decisive differences between the two scenarios occur: whereas scenario 1 may also require monitoring descendants of edited embryos, scenario 2 at most requires monitoring edited individuals. This also seems to make scenario 2 prima facie favorable over scenario 1.

#### Legal perspective: what are the requirements for legitimate germline editing interventions?

From a legal perspective, as long as arguments for a categorical prohibition of human germline editing or, conversely, for an unrestricted permissibility of germline therapies cannot be convincingly established, the legitimacy of such interventions should depend on whether certain strict substantive and procedural requirements are met. In fact, reactions regarding the recent announcement of the birth of genome edited twins [8] have clearly shown the need for normative standards to be strictly complied with in cases of human germline therapies. The object and purpose of such strict substantive and procedural requirements would be the protection of life and health (or physical integrity respectively) and related rights to self-determination of edited embryos, the resulting human beings and its descendants as well as of mothers carrying edited embryos. The lawmaker would have to balance these legal concerns in light of the precautionary principle while taking into account the interests of the international community of states regarding the human genome as “heritage of humanity” [38]. Against this background, for the time being, the following substantive and procedural requirements seem not to be excessively restrictive or unproportional and may, therefore, considered justified.

Concerning substantive requirements, it should be laid down, e.g., that germline interventions are limited to the treatment or prevention of certain serious, hitherto incurable hereditary diseases (such as CF) only and that germline interventions for other, e.g. enhancing or eugenic,^5^ purposes are to be prohibited. Relevant serious diseases could be defined in an abstract way or classified in a list of either exhaustive (i.e. static) or exemplary (i.e. dynamic) character. Compiling and updating such lists might be the legislators’ task or, on the basis of legislatively delegated powers, the task of an administrative authority or of a special committee composed of relevant stakeholders (e.g. scientists, ethicists, lawyers, medical doctors, patient groups).

An additional substantive requirement should be that (preemptive) therapeutic effects, i.e. the cure or prevention of hereditary diseases, are unambiguous and the overall advantage for embryos’ and their offspring’s health is unequivocal. The latter should imply that there are also no negative side effects such as a higher susceptibility to other kinds of diseases.

The permissibility of germline interventions should depend, in addition, on the criterion of necessity. For example, human germline modification might not be necessary in this regard, if an equally effective (preemptive) therapy is available being less intrusive, i.e. not requiring intervening into the germline [51]. Furthermore, with regard to unpredictable long-term effects, a particular germline therapy affecting future generations could be considered unnecessary if a “one-generation therapy” (scenario 2) was available.

Moreover, any clinical application of germline editing should be preceded by rigorous preclinical scientific testing and evaluation using in vitro and in vivo animal models. A current legislative hindrance of clinical trials would be, at least in the EU, that both the EU’s Clinical Trials Directive 2001/20/EC and the new EU’s Clinical Trials Regulation (EU) No. 536/2014 prohibit “gene therapy clinical trials […] which result in modifications to the subject’s germline genetic identity” (Article 90(2) Regulation (EU) No. 536/2014; similarly Article 9(6)(2) Directive 2001/20/EC). However, any form of germline editing in clinical trials would necessarily modify the genetic identity of the respective trial subjects. In line with its positive obligations to protect human life and health, the Union legislator, therefore, might be obliged to review and possibly modify the prohibition laid down in the Clinical Trials Regulation so as to permit clinical germline therapy trials, if they could result in safe therapies as in our scenarios 1 and 2.

In addition, germline interventions, as does any therapeutic intervention, require consent. Obviously, however, embryos are not able to consent to germline interventions [52]. Instead, (future) parents of such embryos could consent to germline treatment. Consent of mothers carrying genetically modified embryos to term will be of particular importance [53]. A more difficult regulatory issue might be whether consent of future generations is required [53] and who should express consent, if, for instance, one-generation genome editing was not applicable. For this purpose, a kind of “trustee” or “custodian” could be established. Since germline editing might affect, albeit over a long period of time, the human gene pool as a whole, such a “trustee” or “custodian” might have to be an international body. These considerations and difficulties speak in favor of only permitting measures as in scenario 2.

The aforementioned substantive requirements would have to be accompanied by procedural requirements. For whether the former are met would have to be reviewed by one or more administrative authorities within the framework of a particular administrative procedure. For the time being, any individual germline intervention should be subject to the requirement of prior authorization. Part of such an authorization procedure could be, e.g., the involvement of an ethics committee with the task to carry out a thorough risk-benefit analysis.

With a view to (international) transparency and traceability, it is advisable to list all authorized germline editing treatments in a registry. In addition, tight monitoring programs should be established in order to survey and control long-term effects of germline interventions.

## Conclusions

Scenario 2 represents a situation in which a clinical application of CRISPR-Cas mediated genome editing interventions in single-cell embryos may be feasible in ethical and legal terms. If the risks of genome interventions can be minimized, future generations excluded from genome editing, and the purpose of the intervention confined to therapeutic measures, there are good reasons to consider the intervention being justified in principle. Further developments on the basis of CRISPR-Cas may provide the means to accomplish the first two requirements. On the other hand, ethical and legal considerations may direct further research on CRISPR-Cas into improving accuracy and elaborating measures to avoid germline transmission to future generations.

Our scenarios reveal a number of questions, which need to be considered from both an ethical and a legal perspective. First, as regards issues of risk and uncertainty, it has to be clarified what risks can legitimately count as acceptable risks. Criteria for the threshold of acceptable risks might be, for instance, that risks are scoring within the risks associated with other established reproductive procedures or that mutations are non-detectable. In any case, arguments for the statutory prohibition of germline interventions would be possibly substantially weakened, if the question of safety was resolved [54]. As far as consequences of germline editing remain uncertain, the precautionary principle entitles states to take preventive measures. However, the exact conditions for applying the precautionary principle as well as adequate precautionary measures have yet to be specified in the context of human germline editing.

Second, as regards responsibilities towards future generations, avoiding any transmission of edited genomes should be persuaded prima facie [55]. Nevertheless, it needs to be clarified whether, and if so in what meaning and consequence, constitutional or international human rights may trigger a positive obligation of the State to protect, e.g., human life and health even of future, not yet existing individuals. From an ethical perspective, it has to be examined whether passing on edited genomes to future generations is to be justified at all, for instance in view of future individuals’ well-being. Similarly, it has to be clarified whether *not* passing on a modified genome to future generations is to be justified in cases where descendants of edited embryos could have benefitted from the intervention.

Third, if no categorical arguments speak against human germline editing, the necessary legitimacy requirements need to be considered. From a legal perspective, several procedural and substantive prerequisites would have to be laid down by law (see Table 1).

**Table 1:** Summary of suggested substantive and procedural legal requirements of germline therapies

**Substantive requirements**	Treatment of, or prevention against, certain serious, hitherto incurable hereditary diseases. Diseases to be defined in an abstract way or identified in a list which could be of an exhaustive (i.e. static) or only exemplary (i.e. dynamic) character
	Interventions for other, e.g. enhancement or eugenic, purposes to be explicitly prohibited
	Acceptable risk of the intervention
	Unambiguous cure or prevention of the hereditary disease and unequivocal overall advantage for the embryo’s and its offspring’s health
	No negative side effects such as a higher susceptibility of the genome edited embryo, or the resulting born human being and its descendants, to other kinds of diseases
	Dependence of the permissibility of germline interventions on necessity
	Preclinical scientific testing and evaluation using in vitro animal and human (e.g. stem cell) models as well as in vivo animal models
**Procedural requirements**	Drawing up, and updating, list of diseases by legislator or, on the basis of legislatively delegated powers, of an administrative authority and/or of a special committee composed of relevant stakeholders (e.g. scientists, ethicists, lawyers, medical doctors, patient groups)
	Consent of the (future) parents of the embryo
	Consent of the mother who will have to carry the genetically modified embryo
	Thorough information about the risks by a medical doctor
	Establishment of international body in form of “trustee” or “custodian” for the purposes of consent of future generations
	Participation in the decision-making process of other institutions such as an ethics committee or a judge

Answering the question of ethical legitimization means considering the conditions under which a germline editing intervention can be understood as (preemptive) therapy, thus legitimizing them. These findings are important prerequisites for the international debate on the ethical and legal justification of germline interventions in the human embryo as well as for the harmonization of international legal standards.

## Data Availability

Not applicable.

## Abbreviations

CCR5: Chemokine Receptor Type 5
CJEU: Court of Justice of the European Union
CFTR: Cystic Fibrosis Transmembrane Conductance Regulator
CRISPR-Cas: Clustered Regularly Interspaced Short Palindromic Repeats-associated
DNA: deoxyribonucleic acid
ECtHR: European Court of Human Rights
EU: European Union
HIV: Human Immunodeficiency Viruses
ICESCR: International Covenant on Economic, Social and Cultural Rights
NIH: National Institutes of Health
PGD: Preimplantation Genetic Diagnosis
RNA: ribonucleic acid

## Footnotes

1 For an overview of regulatory approaches to germline editing in various states see [14, 22].

2 Cf., however, the recent judgment of the Court of Justice of the European Union (CJEU) concerning mutagenesis through genome editing techniques, in which the Court held (with a view to current knowledge as stipulated by the referring national court) that “the risks linked to the use of those new techniques/methods of mutagenesis [such as CRISPR-Cas] might prove to be similar to those which result from the production and release of a [genetically modified organism] through transgenesis” [27].

3 Interestingly, in [27], i.e. in its judgment on mutagenesis through genome editing, the Court did not adhere to its own standards when it self-reliantly considered, without referring to any scientific source, that the precautionary principle was applicable due to “risks for the environment or human health linked to the use of new techniques/methods of mutagenesis [such as CRISPR-Cas] […that] might be similar to those which result from the production and release of a [genetically modified organism] through transgenesis”.

4 Cf [38]., according to which the “human genome” is “the heritage of humanity” (albeit “[i]n a symbolic sense” only).

5 In that regard, it is interesting to note that the European Union (EU) Charter of Fundamental Rights has established an explicit “prohibition of eugenic practices” in its Article 3(2)(b) [50], which may be understood as a bioethical consensus among the EU Member States. In fact, the German legislator, e.g., had already warned as early as in 1989 that dangers of abuse of germline interventions, in particular, for purposes of “human breeding”, were obvious [20].
